# The thyrocervical trunk: an analysis of its morphology and variations

**DOI:** 10.1007/s12565-022-00692-5

**Published:** 2022-11-09

**Authors:** Patryk Ostrowski, Michał Bonczar, Kyrylo Shafarenko, Daniel Rams, Martyna Dziedzic, Kamil Gabryszuk, Michał Zarzecki, Wadim Wojciechowski, Jerzy Walocha, Mateusz Koziej

**Affiliations:** 1 Department of Anatomy, Jagiellonian University Medical College, Cracow, Poland; 2 The Lower Silesian Center of Hand Surgery and Aesthetic Medicine, Chiroplastica, Wrocław, Poland; 3 Department of Radiology, Jagiellonian University Medical College, Cracow, Poland

**Keywords:** Thyrocervical trunk, Subclavian artery, Anatomy

## Abstract

The number of studies on the variations of the branching of the TT is scarce, and those works that treat about the different types of the said trunk are oftentimes inconsistent. Therefore, the authors of the present study would like to propose a set of five types of TT, which were created based on observations of 41 computed tomography angiographies (82 TTs). To establish the anatomical variations, their prevalence, and morphometrical data regarding the TT and its branches, a retrospective study was performed. The results of 55 consecutive patients who underwent neck and thoracic computed tomography angiography (CTA) were analyzed. The analysis was performed on a total of 82 TTs of 41 patients, aged 15 to 82 years (mean age: 46 years; SD: 18.4), of which 16 (39.0%) were females, and 25 (61.0%) were males. Initially, 11 types of variations were evaluated, of which types 1–4 constituted 89.0%. Furthermore, a new method of classification of the anatomical variations of the TTs has been established. In this study, the variety of the branching and morphology of the TT was presented, proposing its novel classification based on the five most commonly prevalent types. Types 1 and 2 were the most common, with a prevalence of 26.8% each. This work also provides physicians with crucial data about the morphology of the TT and its branches, which can surely be of use when performing endovascular or reconstructive procedures in the cervical region.

## Introduction

The thyrocervical trunk (TT) arises from the anterosuperior aspect of the first part of the subclavian artery (SA), medial to the anterior scalene muscle. Anatomy textbooks describe the TT as having four branches, with the largest and most important being the inferior thyroid artery (ITA). The other branches of TT are the ascending cervical artery (ACA), the suprascapular artery (SSA), and the transverse cervical artery (TCA) (Moore et al. 2017).

Variations of the arterial system are frequently observed by medical professionals of many distinct specialties worldwide and oftentimes influence the daily clinical practice in the form of treatment options (Żytkowski et al. 2021; Bonczar et al. 2022b, a). Lischka et al. 1982 reported on the variability of TT and the dorsal scapular artery in 1982, showing the latter to originate from the TT, or the TCA, in 18% of the cases on the left and 29% on the right side. The internal thoracic artery is said to originate from the SA. However, there have been reports of the internal thoracic artery arising from the TT instead (Lischka et al. 1982; Cigali et al. 2008). In many cases, the branches of the TT do not originate directly from the said stem but rather form common trunks or originate from each other (Lischka et al. 1982; Pretterklieber and Pretterklieber 2020), e.g., ACA arising predominantly from the ITA (60%) (Lischka et al. 1982).

Pseudoaneurysms and arteriovenous fistulae of the TT and its branches are possible complications of traumatic or iatrogenic arterial injuries to the said vessels. Most of these result from an iatrogenic needle puncture of the TT whilst performing internal jugular vein catheterization (Hamamoto et al. 2014). The branches originating from the TT are often used as pedicles for numerous flaps, such as the extended supraclavicular fasciocutaneous island flap, which is based on the TCA (Chen et al. 2010). Therefore, a thorough understanding of the morphology and variations of the TT is of a great importance in cervical endovascular and plastic surgery procedures.

Unfortunately, the number of studies on the variations of the branching of the TT is scarce, and those works that treat about the different types of the said trunk are oftentimes inconsistent. Therefore, the authors of the present study would like to propose a set of five types of TT, which were created based on observations of 41 computed tomography angiographies (82 TTs). Additionally, a thorough analysis of the morphology of the trunk and its branches was conducted. The main goal of this paper was to provide an up-to-date extensive description of this highly variable structure and, with that knowledge, aid in diminishing potential surgical complications associated with the TT and its branches.

### Patients and methods

#### Bioethical committee

The research protocol was submitted for assessment and approved by the Bioethical Committee of the Jagiellonian University, Cracow, Poland (1072.6120.51.2022). Further stages of the study were carried out in accordance with the approved guidelines.

#### Study group

To establish the anatomical variations, their prevalence, and morphometrical data regarding the TT and its branches, a retrospective study was performed. The results of 55 consecutive patients who underwent neck and thoracic computed tomography angiography (CTA) were analyzed in the Department of Radiology of the Jagiellonian University Medical College, Cracow, Poland, in March 2022. The result of each patient was analyzed bilaterally. Therefore, a total of 110 TTs were initially evaluated. Exclusion criteria were set as follows: (1) neck or/and thoracic trauma affecting the course of the TT and/or its initial branches, (2) significant artifacts that prevented accurate and precise imaging and/or measurement of the TT and/or its initial branches, (3) low-quality and illegible images, (4) significant lack of filling the whole arterial system with contrast and (5) significant influence of other structures, like anterior scalene muscle, on the course of the TT. Defects, which met the exclusion criteria, but included only one side of the CT, without interference with the contralateral side, did not disqualify the whole CT but only the affected side. Hence, of the initial 110, a total of 28 TTs were excluded to minimalize a potential bias. Of the 28, a great majority (*n* = 25) were excluded due to significant artifacts. The remainder (*n* = 3) were excluded due to a significant lack of contrast filling the entire arterial system. Eventually, forty-one patients met the required criteria. All patients had their TTs visualized bilaterally, which gave a total of 82 TTs in the final analysis.

#### Results acquisition

All head and neck CTA were performed on a 128-slice scanner CT (Philips Ingenuity CT, Philips Healthcare). The main CT imaging parameters were as follows: collimation/increment: 0.625/0.3 mm; tube current: 120 mAs; field of view: 210 mm; matrix size: 512 × 512.

All of the patients received intravenous administration of contrast material at a dose of 1 mL/kg (standard dose). A non-ionic contrast medium (CM) containing 350 mg of iodine per mL was used (Jowersol 741 mg/mL, Optiray^®^, Guerbet, France). CT data acquisition was triggered using a real-time bolus-tracking technique (Philips Healthcare) with the region of interest (ROI) placed in the ascending aorta. The CM was intravenously injected using a power injector at a flow rate of 5 mL/s. This was immediately followed by the injection of 40 mL of saline solution at the same flow rate. Following injection of CM and saline, image acquisition was automatically started with a 2 s delay when the attenuation trigger value reached a threshold of 120 Hounsfield units (HU). Scanning was performed in the caudocranial direction. The CTA examination was started at the level of the aortic arch up to the circle of Willis.

The CTAs were analyzed on a dedicated workstation in the Anatomical Department of Jagiellonian University Medical College, Cracow, Poland. To ensure the highest possible quality of the visualizations and measurements and minimize potential bias, Materialise Mimics Medical version 21.0 software (Materialise NV, Leuven, Belgium) software was used. 3-dimensional (3D) reconstructions of each scan were developed, employing a set of settings, severally adjusted to each scan. A volume rendering opacity range oscillated from 25 to 80 HU for the lower limit and up to 3070 HU for a higher limit. The range was individually adjusted to each TT after a visual investigation.

#### Evaluation and measurements

**Fig. 1:**
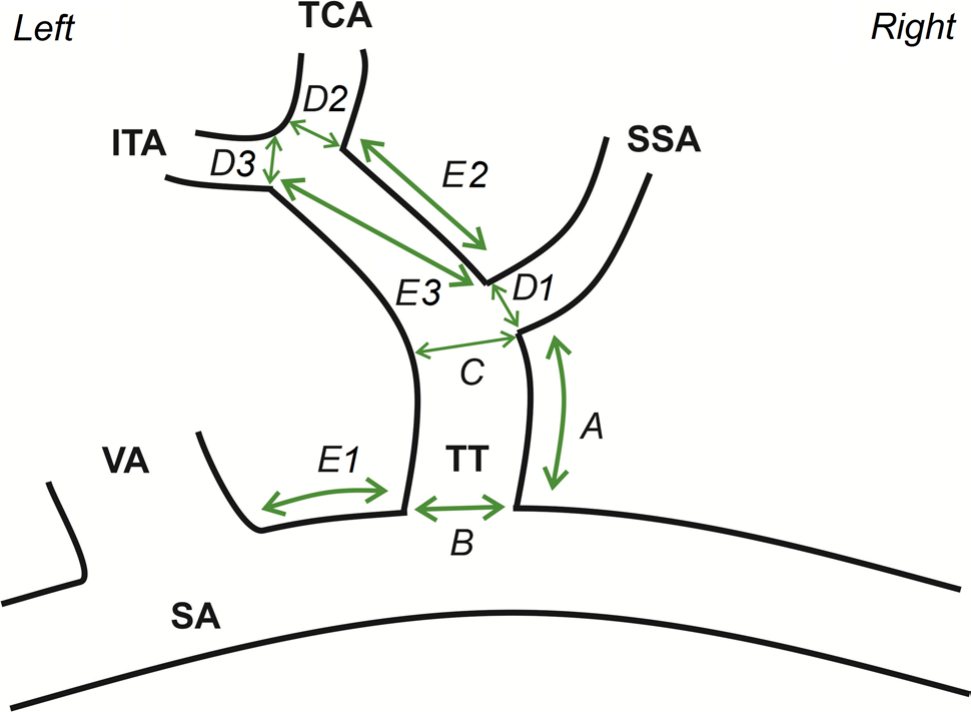
Scheme, illustrating collected measurements on an exemplar thyrocervical trunk (TT). *SA* subclavian artery, *VA* vertebral artery, *SSA* suprascapular artery, *TCA* transverse cervical artery, *ITA* inferior thyroid artery. **A** TT length, **B** TT maximal diameter and ostial area in the start point, **C** TT maximal diameter and ostial area in the endpoint, **D1** maximal diameter and ostial area of the SSA at its origin, **D2** maximal diameter and ostial area of the TCA at its origin, **D3** maximal diameter and ostial area of the ITA at its origin, **E1** shortest distance between the VA and the TT, **E2** shortest distance between the SSA and the TCA, **E3** shortest distance between the SSA and the ITA

At the beginning of each evaluation, the authors ensured that each TT, its branches, and its close anatomical area were fully visualized. Subsequently, each branch of the TT was identified by following its course. The direction of the TT and a set of its branches with their arrangement was evaluated and descriptively noted. Afterward, a set of measurements was executed on each TT. The literature lacks a clear and unequivocal definition of TT and how its length should be measured; thus, the authors decided that the TT length will be established as the shortest distance followed over the surface of the TT (from the SA to the branching off point of its first branch). Therefore, the authors recognize the TT as an arterial branch of the SA, which ends at the sprouting point of its first branch. Therefore, maximal diameter and ostial area were also measured at both the origin and endpoints of the TT. The distance between the TT and the vertebral artery (over the surface of the SA) was also established. Maximal diameters and ostial areas of each TTs branches were measured. Additionally, the shortest distance between each TT branch was set. Figure 1 presents the measurement methods as utilized in this study.

### Statistical analysis

Statistical analysis was performed with SATISTICA v13.1 (StatSoft Inc., Tulsa, OK, USA). Frequencies and percentages presented qualitative features. The Shapiro–Wilk test was used to assess the normal distribution. Quantitative characteristics were presented by medians and upper and lower quartiles (UQ, LQ), as well as means and standard deviation (SD), depending on the verified normality of the data. Statistical significance was defined as *p* < 0.05. U Mann–Whitney and Wilcoxon signed-rank tests were used to establish potential differences between groups. Spearman's rank correlation coefficient was used to determine possible correlations between the parameters.

## Results

The analysis was performed on a total of 82 TTs of 41 patients, in age from 15 to 82 years old (mean age: 46 years old; SD: 18.4), of which 16 (39.0%) were female, and 25 (61.0%) were males. All subsequent results are presented in relation to the number of TTs instead of patients.

**Fig. 2:**
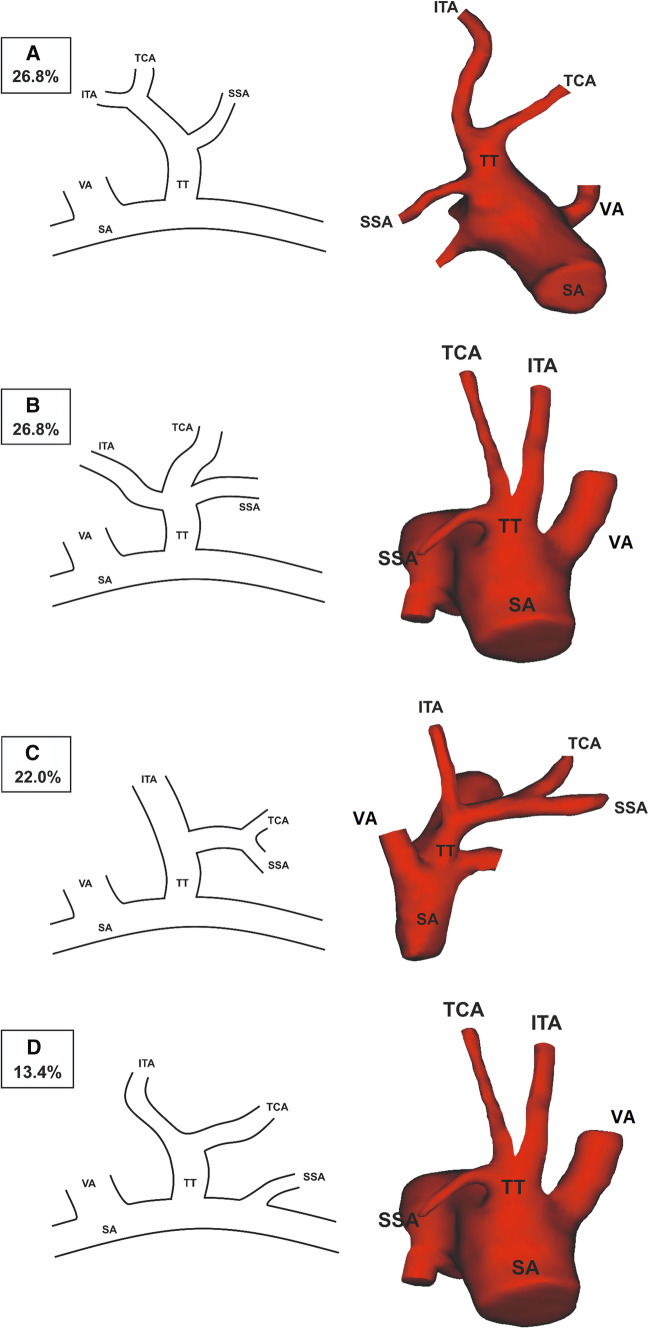
Schemes and 3-dimensional computed topographical angiography reconstructions showing each of the most common types of thyrocervical trunk. **A** Type 1. **B** Type 2. **C** Type 3. **D** Type 4. *SA* subclavian artery, *VA* vertebral artery, *SSA* suprascapular artery, *TCA* transverse cervical artery, *ITA* inferior thyroid artery

**Fig. 3:**
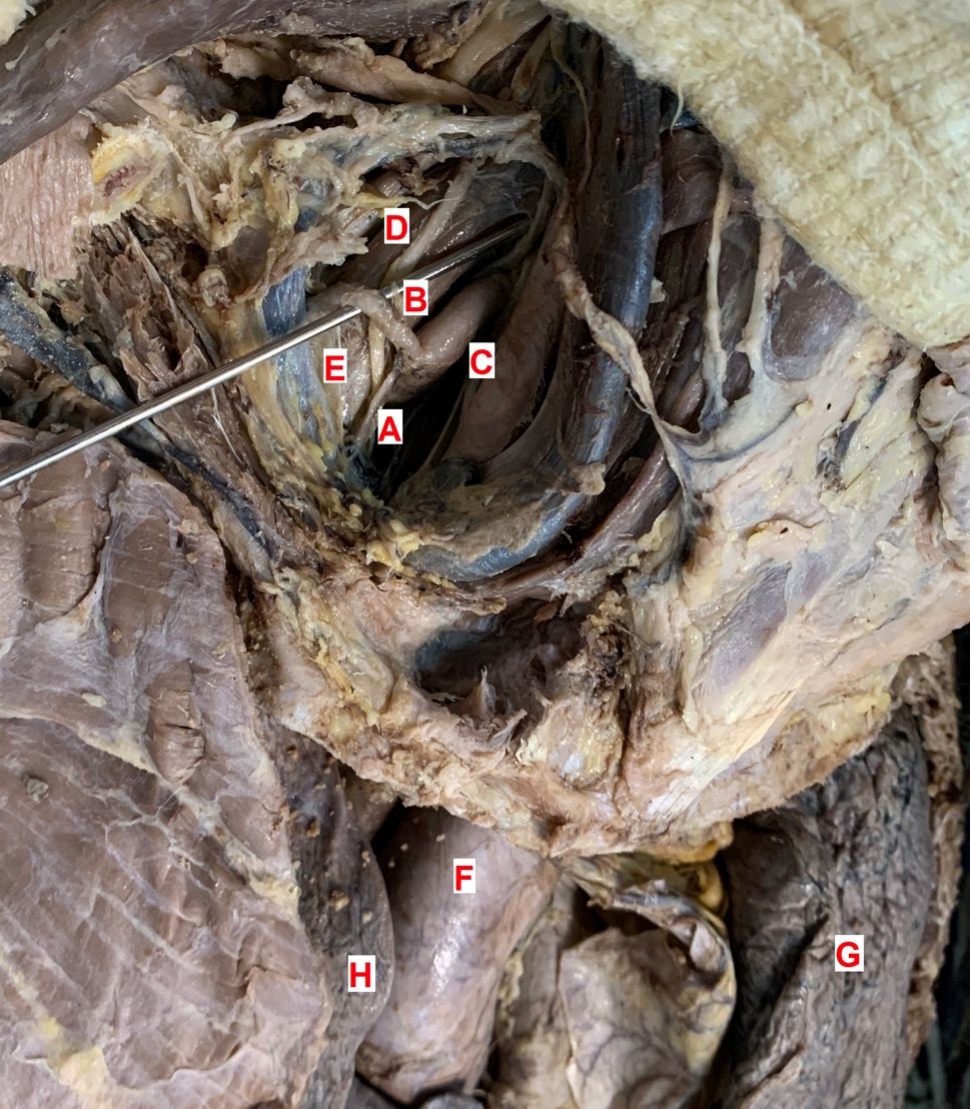
Picture of a cadavers' thyrocervical trunk and its close anatomical area. **A**Thyrocervical trunk. **B** Suprascapular artery. **C** Inferior thyroid artery. **D** Phrenic nerve. **E** Anterior scalene muscle.** F**

The branching variation of each TT was deeply analyzed. Initially, 11 variations types were evaluated, of which types 1–4 constituted 89.0%. Therefore, in response to the literature lacks, a classification method of the TTs was set and consists of 5 different types. The five types were set as follows: (1) Type 1: TT originated from the SA. TT divided into SSA, which sprouted as the first branch, and a further common trunk for ITA and TCA; (2) Type 2: TT originated from the SA. TT divided into ITA, SSA, and TCA without a common trunk for any of the arteries; (3) Type 3: TT originated from the SA. TT divided into a further common trunk for SSA and TCA, which sprouted as the first branch, and an ITA; (4) Type 4: TT originated from the SA. TT divided into ITA and TCA; (5) Type 5: Any other arrangement. Figure 2 depicts schematic diagrams and 3-dimensional CTA reconstructions of each described type of TT. Types were numbered concerning their prevalence. The most common types were found to be types 1 and 2, with a prevalence of 26.8%. Additionally, each TTs direction was evaluated. In the majority of the cases (62.2%), the TT branched off from the SA in an anterior direction. Table 1 presents the qualitative result in detail.(Fig. 3)

**Table 1:** Qualitative results of the data analysis

Category	N	Percentage
Patients’ sex		
Female	32	39.0%
Male	50	61.0%
Types		
Type 1	22	26.8%
Type 2	22	26.8%
Type 3	18	22.0%
Type 4	11	13.4%
Type 5	9	11.0%
The direction of the TT		
Anterior	51	62.2%
Superior	19	23.2%
Anterior and Superior	11	13.4%
Anterior and Inferior	1	1.2%

*TT* thyrocervical trunk. The five types are set as follows: (1) Type 1: TT originates from the SA TT divides into SSA, which departs as a first branch, and a further common trunk for ITA and TCA; (2) Type 2: TT originates from the SA. TT divides into ITA, SSA, and TCA without a common trunk for any of the arteries; (3) Type 3: TT originates from the SA. TT divides into a further common trunk for SSA and TCA, which departs as a first branch, and an ITA; (4) Type 4: TT originates from the SA. TT divides into ITA and TCA; (5) Type 5: Any other arrangement

The TT median maximal diameter at origin was 4.5 mm (LQ: 3.8; UQ: 5.8). The median TT ostial area at origin was 12.7 mm^2^ (LQ: 9.4; UQ: 20.8). The median maximal diameter at endpoint of the TT was found to be 3.8 mm (LQ: 3.5; UQ: 4.8). The median ostial area at endpoint of the TT was found to be 9.8 mm^2^ (LQ: 7.6; UQ: 14.1). According to the results of the sign tests, a statistically significant difference (*p* < 0.05) of both maximal diameters and ostial areas was established between the origin and endpoints of the TT. The detailed results are presented in Table 2.

**Table 2:** Results of the measurements

Category	Median	LQ	HQ	Minimum	Maximum	Mean	SD
TT maximal diameter at the startpoint [mm]	4.5	3.8	5.8	2.5	11.6	5.0	1.7
TT ostial area at the startpoint [mm^2^]	12.7	9.4	20.8	4.3	94.5	17.2	13.3
TT maximal diameter at the endpoint [mm]	3.8	3.5	4.8	2.4	8.7	4.3	1.4
TT ostial area at the endpoint [mm^2^]	9.8	7.6	14.1	2.4	50.8	12.3	8.7
TT length [mm]	4.5	3.3	6.7	1.1	14.9	5.3	3.0
Distance between TT and VA [mm]	7.8	5.1	10.4	1.7	39.9	8.4	5.2
ITA maximal diameter [mm]	2.6	2.1	2.9	1.0	5.0	2.6	0.67
ITA ostial area [mm^2^]	4.6	2.8	6.0	0.71	13.2	4.7	2.5
SSA maximal diameter [mm]	2.2	1.8	2.8	0.85	3.6	2.3	0.65
SSA ostial area [mm^2^]	3.4	2.1	4.8	0.55	8.8	3.6	1.9
TCA maximal diameter [mm]	2.2	2.0	2.7	1.1	4.6	2.4	0.66
TCA ostial area [mm^2^]	3.4	2.4	5.2	0.57	10.8	3.9	2.1
Distance between TCA and SSA [mm]	6.0	4.4	14.6	1.6	50.2	10.3	9.4
Distance between ITA and TCA [mm]	6.5	4.2	11.2	0.8	29.6	8.5	6.3
Distance between ITA and SSA [mm]	10.3	7.3	14.5	1.4	56.0	12.5	9.2

*LQ* lower quartile, *HQ* higher quartile, *SD* standard deviation, *TT* thyrocervical trunk, *VA* vertebral artery, *ITA* inferior thyroid artery, *SSA* suprascapular artery, *TCA* transverse cervical artery

Potential sexual dimorphism was also evaluated. Statistically significant differences between females and males were established regarding (1) maximal diameter of the TT at origin (*p* = 0.02); (2) ostial area of the TT at origin (*p* < 0.001); (3) maximal diameter of the TT at endpoint (*p* < 0.001); (4) ostial area of the TT at endpoint (*p* < 0.001); (5) maximal diameter of the ITA, branching off from the TT (*p* < 0.001); (6) ostial area of the ITA, branching off from the TT (*p* < 0.001); (7) ostial area of the SSA, branching off from the TT (*p* = 0.02); (8) maximal diameter of the TCA, branching off from the TT (*p* = 0.02); (9) ostial area of the TCA, branching off from the TT (*p* < 0.001). There were no statistically significant differences in other groups. Detailed results concerning sex are gathered in Table 3.

**Table 3:** Results of the measurements concerning the sex

Category	Sex	Median	LQ	HQ	Minimum	Maximum	Mean	SD	*P* value
TT maximal diameter at the startpoint [mm]	Female	4.1	3.6	5.1	2.9	6.9	4.4	1.0	0.02
	Male	4.6	3.9	6.4	2.5	11.6	5.3	1.9	
TT ostial area at the startpoint [mm^2^]	Female	10.7	8.2	14.9	6.2	31.9	12.4	6.1	0.00
	Male	14.1	10.6	25.8	4.3	94.5	20.4	15.6	
TT maximal diameter at the endpoint [mm]	Female	3.6	3.2	4.1	2.4	5.8	3.7	0.8	0.00
	Male	4.3	3.6	5.4	2.4	8.7	4.7	1.5	
TT ostial area at the endpoint [mm^2^]	Female	8.2	6.7	10.2	2.4	20.3	8.6	3.4	0.00
	Male	11.2	8.4	18.3	3.2	50.8	14.7	10.1	
TT length [mm]	Female	3.8	3.2	5.3	1.1	9.9	4.6	2.4	0.06
	Male	5.3	3.6	6.8	1.7	14.9	5.8	3.2	
Distance between TT and VA [mm]	Female	6.4	4.4	9.9	2.1	16.5	7.5	3.9	0.29
	Male	8.1	5.2	11.4	1.7	39.9	9.0	5.9	
ITA maximal diameter [mm]	Female	2.2	2.0	2.7	1.0	3.3	2.3	0.5	0.00
	Male	2.7	2.4	3.0	1.2	5.0	2.7	0.7	
ITA ostial area [mm^2^]	Female	3.1	2.3	4.7	0.7	7.4	3.5	1.7	0.00
	Male	5.4	3.9	6.7	0.9	13.2	5.4	2.6	
SSA maximal diameter [mm]	Female	2.0	1.7	2.6	1.1	3.5	2.1	0.6	0.06
	Male	2.4	1.9	3.0	0.9	3.6	2.4	0.7	
SSA ostial area [mm^2^]	Female	2.2	1.9	4.0	0.9	7.3	2.9	1.6	0.02
	Male	3.8	2.6	5.2	0.6	8.8	4.0	2.0	
TCA maximal diameter [mm]	Female	2.1	1.9	2.5	1.1	4.4	2.2	0.6	0.02
	Male	2.5	2.0	2.9	1.2	4.6	2.5	0.7	
TCA ostial area [mm^2^]	Female	2.9	2.1	4.2	0.6	5.9	3.0	1.4	0.00
	Male	3.7	2.7	6.4	0.8	10.8	4.5	2.2	
Distance between TCA and SSA [mm]	Female	5.9	4.3	14.6	1.6	50.2	11.3	12.3	0.85
	Male	6.0	4.4	14.9	1.6	30.6	9.5	7.0	
Distance between ITA and TCA [mm]	Female	6.7	3.8	10.6	0.8	25.3	8.0	6.4	0.53
	Male	6.4	4.3	12.2	1.9	29.6	8.8	6.2	
Distance between ITA and SSA [mm]	Female	8.9	5.8	14.1	1.4	56.0	12.9	12.1	0.26
	Male	11.3	7.9	14.8	2.0	41.8	12.2	6.6	

*LQ* lower quartile, *HQ* higher quartile, *SD* standard deviation, *TT* thyrocervical trunk, *VA* vertebral artery, *ITA* inferior thyroid artery, *SSA* suprascapular artery, *TCA* transverse cervical artery

Additionally, potential differences in the dimensions regarding the patients’ side and direction of the course of TT were analyzed. There were no statistically significant differences (*p* > 0.05) regarding patients’ side and direction of the TT in any category. Co-occurrence of the four main types was established and the results are gathered in Table 4.

**Table 4:** The percentages of co-occurrence of the four main types in the same patient

Co-occurrence	Type 1	Type 2	Type 3	Type 4
Type 1	8.33%			
Type 2	0.00%	8.33%		
Type 3	8.33%	2.78%	11.11%	
Type 4	0.00%	2.78%	8.33%	0.00%

Potential correlations between the age of the patient and each dimension were analyzed. The correlations between patients’ age and (1) maximal diameter of the TT at origin (*R* = 0.23; *p* = 0.03), (2) ostial area of the TT at origin (*R* = 0.26; *p* = 0.02), (3) distance between the vertebral artery and the TT (*R* = 0.25; *p* = 0.03), and (4) ostial area of the internal thoracic artery, branching off from the TT (*R* = − 0.64; *p* = 0.04) were obtained. No other, statistically significant correlations were established.

## Discussion

According to Hamilton et al. (Hamilton et al. 1945), the dorsolateral somatic intersegmental arteries sprout from the dorsal aorta and are responsible for the arterial supply to somites, i.e., vessels of the body wall during the human ontogeny. The said vessels run transversely and have a subsequent division into the dorsal branches (that are principally responsible for the vascularization of the spinal cord), as well as the ventrolateral branches (with the ventral supplying the body wall and joining with the contralateral ones, while the lateral serve as axial limb arteries) (Arey 1942; Hamilton et al. 1945). In addition, both main branches have longitudinal interconnections with the dorsolateral arteries located superiorly and inferiorly to the artery in question. These are in the form of precostal, postcostal, and post-transverse longitudinal anastomoses (Hamilton et al. 1945). Although the vertebral artery originates from the postcostal interconnections of the arteries in the cervical and upper thoracic regions (Schmeidel 1932), the precostal anastomoses give rise namely to the thyrocervical trunk analyzed in the present study, as well as to the superior intercostal stem (Arey 1942; Hamilton et al. 1945). Lastly, the persistence of the post-transverse interconnections forms the deep cervical artery (Hamilton et al. 1945).

The first extensive review of the morphology and variations of the TT was conducted in 1982 by Lischka et al. (Lischka et al. 1982). The study consisted of a cadaveric analysis of the TT and the dorsal scapular artery. The authors of the said article divided the TT into three major types, namely type I: a prominent ITA that gives off slender collaterals for the omocervical supply (TCA, SSA, etc.); type II: a short common stem of ITA and the omocervical vessels (TCA, SSA, etc.) representing a true thyrocervical trunk; and type III: ITA and the omocervical vessels do not form a common trunk but arise separately from the subclavian artery. The aforementioned types are less detailed than the classification proposed in the present study. Garcia et al. (Pérez-García et al. 2018) described numerous interventional radiological procedures performed through the TT, such as embolization of paragangliomas, with an additional discussion about the vascular anatomy of the TT and its variants. In the said study, eight endovascular procedures through the TT were described, with an illustration presenting six anatomical variations of the TT (A to F). However, these types were not extensively described, and the reported prevalence of the types is limited due to the low number of specimens.

To the best knowledge of the authors, the current study is the first to evaluate the morphology and variations of the TT using CTA. Due to inconsistencies in the classification of this structure, the study presented a set of five types of TT branching patterns. The data in this study shows that the types of TT are dominated by type 1–4 (89.02%). The TT consisted of the ITA, SSA, and TCA in types 1–3, showing that these branches quite consistently originate from the TT. However, type 4 (13.41%) represents a TT where the SSA does not originate from the said trunk but rather sprouts directly from the SA or other surrounding arterial entities. Interestingly, in some cases, the SSA was not found at all. Nevertheless, this type is relatively less prevalent than types 1–3. Anatomical textbooks describe the TT as giving off four branches; the ITA, TCA, SSA, and the ACA (Moore et al. 2017). However, our study shows that the ACA is not a consistent branch of the TT, found to sprout from it only in a single case (1.22%). The remainder of the ACAs originated from the ITA and had a usual course upwards in the cervical region. Hence, the authors would like to suggest that the TT is the origin point of the three main branches (namely the ITA, TCA, and SSA), instead of the classically described four (with the addition of the ACA to the former).

In the present study, type 5 represents a TT with any other vascular arrangement than those presented in types 1–4. Numerous variations of the TT make up type 5 in this classification. The internal thoracic artery originating from the TT was observed four times, in contrast to its typical sprouting point at the SA. Additionally, there have been other reports of the internal thoracic artery originating from the TT in the literature (Lischka et al. 1982; Cigali et al. 2008; Pretterklieber and Pretterklieber 2020). Different variants of types 1–4 were also observed, such as type 3 with a common trunk formed by the ITA and the SSA, rather than the TCA and the SSA. Numerous variations of the vertebral artery have been reported in the literature (Kahn et al. 2018; Onrat et al. 2021). A case of a vertebral artery arising from the TT was noted when conducting the present study. This variation has also been described by Strub et al. (2006). Other additional branches than the aforementioned ones were also discovered. The ACA was observed to originate from the TT only once. However, Lischka et al. (Lischka et al. 1982) presented the ACA arising from the TCA in 17% of the cases. This origin of the ACA was not observed when conducting the present study. Furthermore, the cited authors reported that the dorsal scapular artery originated from the TT or the TCA in 18% of the cases on the left and 29% on the right side (Lischka et al. 1982). The authors of this study did not observe any dorsal scapular arteries arising from the TT or the proximal parts of the TCA. However, the further branching pattern of the TCA was not significantly analyzed. Therefore, there is a possibility that the dorsal scapular artery can, in some instances, arise from the TCA rather than the SA, and this topic requires further investigation.

Pseudoaneurysms and arteriovenous fistulae of the TT and its branches are rare complications of iatrogenic arterial injuries. Most of these complications are caused by needle puncture of the TT when trying to catheterize the internal jugular vein (Hamamoto et al. 2014). A pseudoaneurysm should generally be treated due to possible complications, including a mass effect involving adjacent structures, pain, and rupture. However, arteriovenous fistulae rarely require intervention because they often resolve spontaneously (Thalhammer et al. 2000). A possible risk factor for an iatrogenic pseudoaneurysm of the TT is using a lower or a lateral approach to the internal jugular vein when performing catheterization (Shield et al. 1975). This study presents a statistically significant sexual dimorphism regarding dimensions of the ostial areas and maximal diameters of the TT and its most common branches. Additionally, correlations between some measurements and age are suggested. The most common anatomical variations of the TT and their prevalence were established, showing relatively compelling variability of this structure. Physicians, especially those who deal with endovascular procedures, such as catheterizations for coronary and intracardiac access through the upper limb, should be familiarized with the detailed and precise anatomical knowledge of this particular area, as it can reduce the risks of potential complications (Criado et al. 2000; Bois et al. 2018).

The TT is an intermediary between interventional vascular radiology and neurovascular radiology because it gives off branches that supply both the cervical and upper limb structures. Perez-Garcia et al. (Pérez-García et al. 2018) presented a study on embolization procedures through the TT. In the said research, they showed how the branches of the TT provide blood supply to the musculoskeletal and nervous structures of the neck and how crucial it is to have knowledge of the variations and anastomosis of the vascular anatomy of the neck-thorax-upper limb region. The most important one might be formed with the cervical spinal cord vascularization through the anterior and posterior spinal arteries and with the brain stem through the vertebral arteries. An et al. (2008) reported a posterior cervical spinal cord infarction case, following TT embolization for hemoptysis. The aforementioned study hypothesized that the posterior spinal cord infarction resulted from occlusion of a singular posterior spinal radicular artery arising from the TT, supplying blood to both sides of the posterior spinal cord at the C5–C7 level.

Knowledge about the branching of the TT can also be of great use in plastic and reconstructive procedures. The main supraclavicular vessel is the TCA, located in the posterior triangle of the neck. It is also the main vascular supply of the supraclavicular fasciocutaneous island flap (SFIF). Chen et al. (2010) described the use of an SFIF to reconstruct head and neck defects after cancer ablation in 24 patients. The pedicled SFIF extended to include the shoulder skin, based on the cutaneous feeder vessels and perforator vessels in the deep fascia of the TCA (Chen et al. 2010). Furthermore, Vinh et al. (2009) conducted a study on the supraclavicular flap, analyzing 103 flaps that were used to reconstruct neck scar contractures. Of the 103 flaps, 97 survived completely (94.2%), demonstrating the reliability of this type of flap (Vinh et al. 2009).

The sternocleidomastoid flap has been a solid option for repairing defects of the floor of the mouth, mandible, and pharynx (Ariyan 1980). Leclere et al. (2012) presented a cadaveric study, comprising 15 donors, on the blood supply of the sternocleidomastoid muscle. The study showed that the lower third of the muscle was always supplied by branches of the TT, most commonly by the SSA (Leclère et al. 2012).

The present study undoubtedly has some limitations. Although the size of the study group used in the current paper is the largest among imaging studies concerning the TT, larger population-based research is still warranted to discern the true prevalence of its variants. Additionally, radiological imaging only allows one to evaluate hemodynamically efficient arteries. Therefore, this can be a relatively big source of bias when assessing anatomical variations of the TT, and other arterial entities. The particular branches of the TT and their anastomosis with surrounding vascular structures should be further investigated in the future due to their potential clinical significance in endovascular and reconstructive procedures.

## Conclusions

In this study, the variety of the branching and morphology of the TT was presented, proposing its novel classification based on the five most commonly prevalent types. Types 1 and 2 were the most common, with a prevalence of 26.8% each. Type 1 consisted of a TT that gives off a SSA, and a common trunk between ITA and TCA. Type 2 presented a TT that gave off these three aforementioned branches directly, without a common trunk for any of them. This work also provides physicians with crucial data about the morphology of the TT and its branches, which can surely be of use when performing endovascular or reconstructive procedures in the cervical region.

## Data Availability

The data that support the findings of this study are available from the corresponding author, upon reasonable request.
